# Use of Deep Learning to Examine the Association of the Built Environment With Prevalence of Neighborhood Adult Obesity

**DOI:** 10.1001/jamanetworkopen.2018.1535

**Published:** 2018-08-31

**Authors:** Adyasha Maharana, Elaine Okanyene Nsoesie

**Affiliations:** 1 Department of Biomedical Informatics and Medical Education, University of Washington, Seattle; 2 Institute for Health Metrics and Evaluation, University of Washington, Seattle

## Abstract

**Importance:**

More than one-third of the adult population in the United States is obese. Obesity has been linked to factors such as genetics, diet, physical activity, and the environment. However, evidence indicating associations between the built environment and obesity has varied across studies and geographical contexts.

**Objective:**

To propose an approach for consistent measurement of the features of the built environment (ie, both natural and modified elements of the physical environment) and its association with obesity prevalence to allow for comparison across studies.

**Design:**

The cross-sectional study was conducted from February 14 through October 31, 2017. A convolutional neural network, a deep learning approach, was applied to approximately 150 000 high-resolution satellite images from Google Static Maps API (application programing interface) to extract features of the built environment in Los Angeles, California; Memphis, Tennessee; San Antonio, Texas; and Seattle (representing Seattle, Tacoma, and Bellevue), Washington. Data on adult obesity prevalence were obtained from the Centers for Disease Control and Prevention’s 500 Cities project. Regression models were used to quantify the association between the features and obesity prevalence across census tracts.

**Main Outcomes and Measures:**

Model-estimated obesity prevalence (obesity defined as body mass index ≥30, calculated as weight in kilograms divided by height in meters squared) based on built environment information.

**Results:**

The study included 1695 census tracts in 6 cities. The age-adjusted obesity prevalence was 18.8% (95% CI, 18.6%-18.9%) for Bellevue, 22.4% (95% CI, 22.3%-22.5%) for Seattle, 30.8% (95% CI, 30.6%-31.0%) for Tacoma, 26.7% (95% CI, 26.7%-26.8%) for Los Angeles, 36.3% (95% CI, 36.2%-36.5%) for Memphis, and 32.9% (95% CI, 32.8%-32.9%) for San Antonio. Features of the built environment explained 64.8% (root mean square error [RMSE], 4.3) of the variation in obesity prevalence across all census tracts. Individually, the variation explained was 55.8% (RMSE, 3.2) for Seattle (213 census tracts), 56.1% (RMSE, 4.2) for Los Angeles (993 census tracts), 73.3% (RMSE, 4.5) for Memphis (178 census tracts), and 61.5% (RMSE, 3.5) for San Antonio (311 census tracts).

**Conclusions and Relevance:**

This study illustrates that convolutional neural networks can be used to automate the extraction of features of the built environment from satellite images for studying health indicators. Understanding the association between specific features of the built environment and obesity prevalence can lead to structural changes that could encourage physical activity and decreases in obesity prevalence.

## Key Points

**Question:**

How can convolutional neural networks assist in the study of the association between the built environment and obesity prevalence?

**Findings:**

In this cross-sectional modeling study of 4 US urban areas, extraction of built environment (ie, both natural and modified elements of the physical environment) information from images using convolutional neural networks and use of that information to assess associations between the built environment and obesity prevalence showed that physical characteristics of a neighborhood (eg, the presence of parks, highways, green streets, crosswalks, diverse housing types) can be associated with variations in obesity prevalence across different neighborhoods.

**Meaning:**

The convolutional neural network approach allows for consistent quantification of the features of the built environment across neighborhoods and comparability across studies and geographic regions.

## Teaser

This cross-sectional modeling study assesses features in the built environment and provides associations with obesity prevalence at the census tract level for 6 US cities.

## Introduction

The Global Burden of Disease study estimates that more than 603 million adults worldwide were obese in 2015.^1^ In the United States, more than one-third of the adult population is obese,^2,3,4^ and 46 states have an estimated adult obesity rate of 25% or more.^5^ Obesity is a complex health issue that has been linked to a myriad of factors, including genetics, demographics, and behavior.^6^ Behavioral traits that encourage unhealthy food choices and a sedentary lifestyle have been associated with features in the social and built environment (ie, both natural and modified elements of the physical environment). The built environment can influence health through the availability of resources, such as housing, activity and recreational spaces, and measures of community design.^7^

Studies^8,9,10,11,12,13,14,15,16,17,18,19,20,21,22,23,24,25,26^ have shown that certain features of the built environment can be associated with obesity and physical activity across different life stages. Research also supports the association between obesity and environmental factors, including walkability, land use, sprawl, area of residence, access to resources (eg, recreational facilities and food outlets), level of deprivation, and perceived safety.^27,28,29,30^ Proximity and access to natural spaces and sidewalks can lead to increased and regular physical activity, especially in urban areas.^31,32,33,34,35^

Despite these associations between obesity and the built environment, inconsistencies have been noted across studies and geographic contexts on the association between specific features of the built environment and obesity prevalence.^28,36,37,38^ These inconsistencies could be due to variations in measures and measurement tools across studies, making it difficult to assess and compare findings.^29^ Furthermore, the process of measuring these features can be costly, time-consuming, and subject to human judgment and bias. Approaches that enable consistent measurement and allow for comparison across studies are needed. Assessing and quantifying the association of the built environment with obesity would be useful for selecting and implementing appropriate community-based interventions and prevention efforts.^4,29^

Herein, we propose a method for comprehensively assessing the association between adult obesity prevalence and the built environment that involves extracting neighborhood physical features from high-resolution satellite imagery using a previously trained (hereafter termed *pretrained*) convolutional neural network (CNN), a deep learning approach. Nguyen et al^39^ used CNNs to classify images of the built environment from Google’s Street View to assess the association between obesity and the presence of crosswalks, building types, and street greenness or landscaping. However, their study did not take full advantage of CNNs for independently discovering features that can be associated with obesity prevalence and was limited to 3 predetermined features. In contrast, we comprehensively assess features in the built environment and demonstrate our approach by providing fine-grained associations with obesity prevalence at the census tract level for 4 US regions. Our approach is also scalable and relies on openly available data and computational tools and can enable comparability across studies.

## Methods

### Obesity Prevalence Data

We used 2014 estimates of annual crude obesity prevalence at the census tract level from the 500 Cities project.^2,40,41^ These estimates were computed from the Behavioral Risk Factor Surveillance System data, wherein the survey respondents are 18 years or older and the body mass index (BMI; calculated as weight in kilograms divided by height in meters squared) threshold for obesity is 30. We selected cities from states with high (Tennessee and Texas) and low (Washington and California) prevalence of obesity.^5^ The 6 cities selected included Los Angeles, California; Memphis, Tennessee; San Antonio, Texas; and Seattle, Tacoma, and Bellevue, Washington. Because Seattle, Tacoma, and Bellevue are neighboring cities with few census tracts, we combined their data into a single data set, hereinafter referred to as Seattle. The study was exempt from institutional review board approval because the research involved the study of existing data and records collected by external parties in such a manner that individuals cannot be identified. This study followed the Strengthening the Reporting of Observational Studies in Epidemiology (STROBE) reporting guideline where applicable.

The analysis consisted of 2 steps. First, we processed satellite images to extract features of the built environment using the CNN and extracted and processed point-of-interest (POI) data. Second, we used elastic net regression to build a parsimonious model to assess the association between the built environment and obesity prevalence.

### Acquiring Satellite Imagery and POI Data

We downloaded images from Google Static Maps API (application programing interface) by providing the geographic center, image dimensions, and zoom level for each image. The zoom level and image dimensions were set to 18 and 400 × 400 pixels, respectively, for the entire data set. For each city, we divided its geographic span into a square grid, where each point is a pair of latitude and longitude values and the grid spacing is approximately 150 m. Further, we used census tract shapefiles to associate each image with its respective census tract and excluded images that were from areas outside the city limits. We used the same square grid to select geographic locations and performed a radial nearby search within an appropriate distance to download POI data through the Google Places of Interest API. Points of interest that were located outside city limits were excluded. We collected a set of 96 unique POI categories, and for each census tract we counted the number of locations associated with each category (eTable 1 in the Supplement). All POI data and satellite images were initially downloaded from February 14 through 28, 2017, and updated during the study period, which lasted through October 31, 2017. Satellite images downloaded through the API are only marked with the date of download represented by a time stamp and watermark at the bottom.

### Image Processing

Convolutional neural networks have achieved groundbreaking success with large data sets in critical computer vision tasks (eg, object recognition, image segmentation) as well as health-related applications (eg, recognition of skin cancer)^42^ and estimating poverty.^43^ Owing to the lack of a large labeled data set for classifying high- and low-obesity regions, we adopted a transfer learning approach (eFigures 1 and 2 in the Supplement), which involves using a pretrained network to extract features of the built environment from our unlabeled data set of nearly 150 000 satellite images. Transfer learning involves fine-tuning the pretrained CNN for a new task (with modification to the output layer) or using the pretrained CNN as a fixed feature extractor combined with linear classifiers or regression models. These approaches have been successfully implemented to perform computer vision tasks that are markedly different from object recognition.^44^

We used the VGG-CNN-F network,^45^ which is composed of 8 layers (5 convolutional and 3 fully connected) and is trained on approximately 1.2 million images from the ImageNet database (a data set of >14 million images used for large-scale visual recognition challenges)^46^ for recognizing objects belonging to 1000 categories.^47^ The network learns to extract gradients, edges, and patterns that aid in object detection. Studies using similar transfer learning approaches^48,49^ have shown that features extracted from networks trained on the ImageNet data are effective at classifying aerial imagery into fine-grained semantic classes of land use (eg, golf courses, bridges, parking lots, buildings, and roads).

**Figure 1.**
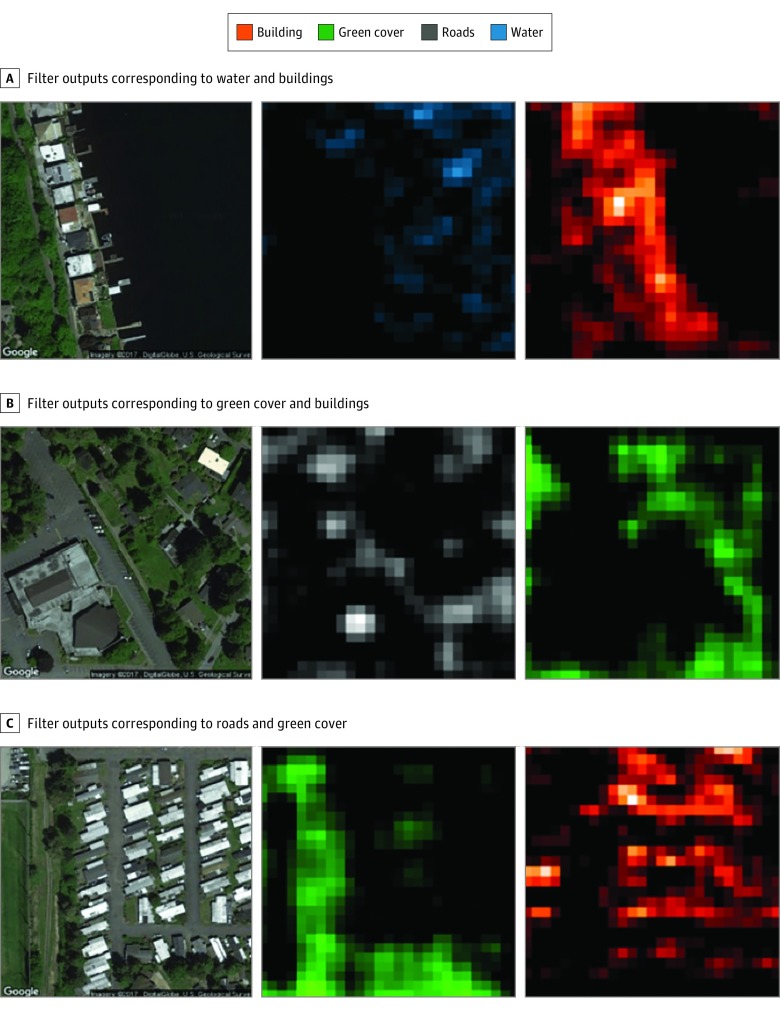
Visualization of Features Identified by the Convolutional Neural Network (CNN) Model The images on the left column are satellite images taken from Google Static Maps API (application programing interface). Images in the middle and right columns are activation maps taken from the second convolutional layer of VGG-CNN-F network after forward pass of the respective satellite images through the network. The CNN understands image by interpreting the output from filters learned during the training phase. The activation maps may not always align exactly with the original image owing to padding of output within the CNN. From Google Static Maps API, DigitalGlobe, US Geological Survey (accessed July 2017).

We collected outputs from the second fully connected layer of the network for each image in our data set.^45,50^ The second fully connected layer has 4096 nodes, each of which has nonlinear connections with all other nodes in the previous and next layers. Each feature vector has 4096 dimensions, corresponding to the output (also termed *activations*) from these nodes. These outputs were further aggregated into mean feature vectors for each census tract by computing the mean from all images belonging to a census tract. We do not link these features to specific elements in the built environment. Rather, these features collectively represent an indicator of the built environment. To investigate whether the CNN can differentiate between built environment features, we made a forward pass through the network for a randomly selected set of images and examined the output maps from convolutional layers of the CNN (Figure 1). We also group image features to illustrate that built environment characteristics in areas with low and high obesity prevalence are distinct (eFigure 3 in the Supplement).

### Statistical Analysis

We applied Elastic Net,^51^ a regularized regression method that eliminates insignificant covariates, preserves correlated variables, and is well suited to the high-dimensional (n = 4096) feature vectors extracted from our image data set. Regularization in Elastic Net guards against overfitting, which is a concern given the high dimensionality of our feature data set. To select an appropriate value for the tuning parameter (λ value), we used cross-validation and selected the value that minimized the mean cross-validated error.

We performed 5-fold cross-validated regression analyses to quantify the following associations: (1) between features of built environment and prevalence of obesity at the census tract level, (2) between density of POIs and prevalence of obesity at the census tract level, and (3) between features of built environment and per capita income obtained from the American Community Survey 2014 five-year estimates at the census tract level (eTable 2 in the Supplement).^52^ We also split the data into 2 random samples, using 1 sample representing 60% of the data in model fitting and the remaining 40% in validation across all analysis. These analyses were performed jointly for all regions and independently for each region. Additional details are available in eMethods in the Supplement.

## Results

We included 1695 census tracts from the 6 cities. Based on 2010 census data, individuals 18 years and older constituted 78.8% (n = 96 410) of the population of Bellevue, 84.6% (n = 515 147) of the population of Seattle, 77.0% (n = 152 760) of the population of Tacoma, 76.9% (n = 2 918 096) of the population of Los Angeles, 74.0% (n = 478 921) of the population of Memphis, and 73.2% (n = 971 407) of the population of San Antonio. The per capita income varied across these cities, with cities such as Bellevue and Seattle having much higher values. Specifically, the corresponding values in US dollars obtained from the American Community Survey 2014 five-year estimates were $50 405 for Bellevue, $44 167 for Seattle, $26 805 for Tacoma, $28 320 for Los Angeles, $21 909 for Memphis, and $22 784 for San Antonio. The cities with the highest per capita income also had the lowest city-level age-adjusted obesity prevalence estimates, at 18.8% (95% CI, 18.6%-18.9%), and 22.4% (95% CI, 22.3%-22.5%), respectively, for Bellevue and Seattle. In contrast, the age-adjusted obesity prevalence was 30.8% (95% CI, 30.6%-31.0%) for Tacoma, 26.7% (95% CI, 26.7%-26.8%) for Los Angeles, 36.3% (95% CI, 36.2%-36.5%) for Memphis, and 32.9% (95% CI, 32.8%-32.9%) for San Antonio.

**Figure 2.**
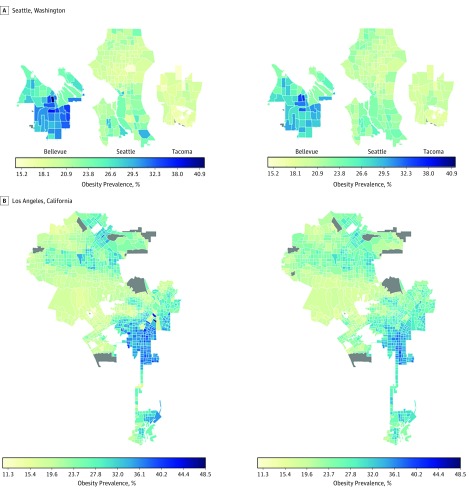
Actual Obesity Prevalence and Cross-Validated Model Estimates of Obesity Prevalence in High-Prevalence Areas Images on the right represent actual obesity prevalence; on the left, cross-validated estimates of obesity prevalence based on features of the built environment extracted from satellite images. Images from the Seattle region include Bellevue, Seattle, and Tacoma. The gray shaded regions do not have data.

**Figure 3.**
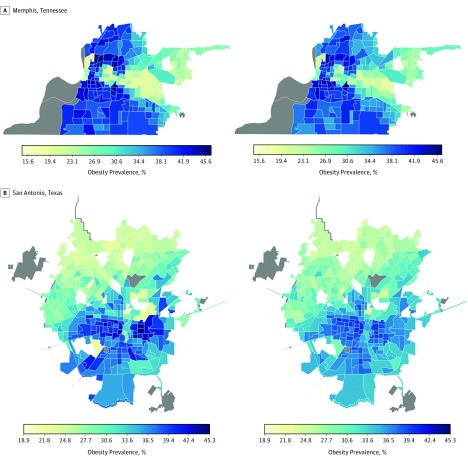
Actual Obesity Prevalence and Cross-Validated Model Estimates of Obesity Prevalence in Low-Prevalence Areas Images on the right represent actual obesity prevalence; on the left, cross-validated estimates of obesity prevalence based on features of the built environment extracted from satellite images. The gray shaded regions do not have data.

Visualization of the outputs from the convolutional layers of the VGG-CNN-F suggests that our model learns to identify features of the environment that have been associated with obesity from the satellite images. Specifically, the CNN captured gradients and edges corresponding to the presence of roads, buildings, trees, water, and land (Figure 1). After regularization, we retained 125 features for all cities combined, 157 for Los Angeles, 79 for Memphis, 69 for San Antonio, and 85 for Seattle. These features of the built environment explained 64.8% (root mean square error [RMSE], 4.3) of the variation in obesity prevalence in out-of-sample estimates across all 1695 census tracts based on the elastic net regression. Individually, our models explained 55.8% (RMSE, 3.2) of the variation in obesity prevalence for Seattle (213 census tracts), 56.1% (RMSE, 4.2) for Los Angeles (993 census tracts), 73.3% (RMSE, 4.5) for Memphis (178 census tracts), and 61.5% (RMSE, 3.5) for San Antonio (311 census tracts) in out-of-sample estimates (Figure 2 and Figure 3 and eFigures 4-11 in the Supplement). Our approach consistently presents a strong association between obesity prevalence and the built environment indicator across all 4 regions, despite varying city and neighborhood values. These high associations between features of the built environment and obesity were achieved without labeling the satellite images or fine-tuning the CNN model to differentiate between images from neighborhoods with high vs low prevalence of obesity.

**Figure 4.**
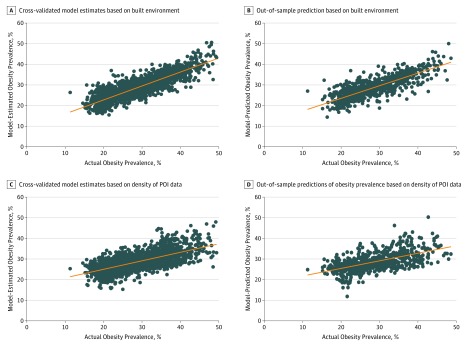
Scatterplots of Model-Estimated and Model-Predicted Obesity Prevalence Plotted Against Actual Obesity Prevalence Built environment information is extracted from satellite images. POI indicates point of interest.

Compared with the features of the built environment, the POI data explained 42.4% (RMSE, 4.3) of the variation in obesity prevalence across all 1695 census tracts in out-of-sample estimates. The variation explained at the regional level was approximately 14.0% (RMSE, 4.5) for the 213 Seattle census tracts, 29.2% (RMSE, 5.4) for the 993 Los Angeles census tracts, 43.0% (RMSE, 4.1) for the 311 San Antonio census tracts, and 43.2% (RMSE, 6.7) for the 178 Memphis census tracts. We illustrate the linear correlation between the actual obesity prevalence and our model-estimated prevalence and compare the findings using the image features and POI data in Figure 4 and eFigure 12 in the Supplement.

One possible explanation of the significant associations between the data on the built environment features and obesity prevalence is that the data might be representative of socioeconomic indicators such as income. Specifically, the variation in per capita income explained by the features of the built environment was 37.6% for the 213 Seattle census tracts, 62.1% for the 993 Los Angeles census tracts, 58.2% for the 311 San Antonio census tracts, and 23.2% for the 178 Memphis census tracts (eFigures 13-16 in the Supplement). These observations suggest that for cities such as Los Angeles and San Antonio, most of the significant association between obesity prevalence and the features of the built environment could potentially be explained by variations in socioeconomic status. This suggestion agrees with a previous study^43^ that used CNNs and satellite imagery to assess the association between the built environment and poverty. However, the inconsistency in the associations across the 4 regions also suggests that features discerned by the CNN might capture additional information not directly linked to socioeconomic indicators.

## Discussion

In this study, we used a CNN, a deep learning approach, to extract data representing features of the built environment from high-resolution satellite images to examine the association between the built environment and the prevalence of obesity across 4 regions. The CNN features capture different aspects of the environment, such as greenery and different housing types, that have been associated with physical activity and obesity. Our results demonstrate a consistent association between the built environment indicator and obesity prevalence across neighborhoods with low and high prevalence of adult obesity. A sampling of the satellite images from various census tracts with high and low prevalence of obesity indicates that these environments have features that have been linked with low and high prevalence of obesity in other studies. For example, we observe neighborhoods with tightly packed houses vs spaced housing (eFigure 11 in the Supplement), neighborhoods close to roadways vs those with smaller streets and crosswalks (eFigure 9 in the Supplement), neighborhoods with much vs less greenery (eFigure 7 in the Supplement), and affluent vs poor neighborhoods (eFigure 5 in the Supplement).

In some instances, our models tended to underestimate obesity prevalence, especially for Seattle, which could be explained by the presence of green spaces in the region and the low variation of obesity prevalence across most of the region. Overestimation of obesity prevalence as observed in some of the high-income census tracts in the east of Memphis could be explained by the presence of features that do not encourage physical activity; however, individuals can more readily afford gym memberships and other recreational facilities. These observations therefore suggest that the features of the built environment can be used in combination with other data sources for monitoring obesity prevalence, and these data could be useful for regions with delayed updates on obesity estimates and for programs focused on reducing obesity.

Our study is important for several reasons. First, although the built environment has been postulated to have an effect on obesity prevalence, studies have produced varying results, partly because measures of the built environment can be subjective, sometimes relying on participant or researcher perceptions. Second, in some studies, neighborhood audits of features of the built environment have heretofore been conducted using costly and time-consuming on-site visits or neighborhood surveys. The development of data algorithms that can automatically process satellite images to create indicators of the built environment would dramatically lower the cost and allow for investigations of the effect of place characteristics on obesity prevalence. Third, our study presents strong and consistent evidence suggesting that the built environment may be a significant indicator of obesity prevalence.

The proportion of variation (*R*^2^) in obesity prevalence explained in studies that report this measure has varied. For example, the percentage of between–census tract variance explained increased from 77% to 87% when built environment variables were added to a model for BMI composed of demographic variables.^53^ However, not all the built environment features (ie, land use mix, subway density, bus stop density, intersection density) were significant. On adding built environment variables (including land-use mix, distribution of fast-food outlets, street connectivity, access to public transportation, and green and open spaces) to a model for overweight and obesity that included individual and neighborhood level covariates, Li et al^54^ observed that the model explained neighborhood-level variation of 0.981. The proportion of variation observed in our analyses could be owing to the comprehensiveness of our approach and possible confounding factors such as socioeconomic status. However, although our findings are likely to be explained at least in part by socioeconomic indicators, such as income, our analyses also suggest that the built environment features more consistently estimate obesity than per capita income across all regions. A possible explanation is that the features extracted include man-made changes to the built environment and natural features (eg, parks and forests) that might not always be indicative or particularly associated with socioeconomic status.

We also demonstrate that the density of POI can also be associated with variations in obesity prevalence, but to a lesser extent when compared with results obtained using the data extracted from satellite images. The POI data displayed higher associations with obesity prevalence for regions with higher obesity prevalence (ie, San Antonio and Memphis) and less so in regions with lower obesity prevalence. Furthermore, some of the most significant variables could be directly associated with health, diet, and exercise (eg, gyms, spas, restaurants, bakeries, supermarkets, bowling alleys), whereas others might be linked to other neighborhood characteristics (eg, natural features, pet stores, recreational vehicle parks). Also, strictly restricting the data to POIs associated with health and exercise resulted in poorer results. Furthermore, the POI data support the results from the satellite imagery analysis; specifically, both analyses suggest stronger associations between the built environment and obesity for San Antonio and Memphis compared with Los Angeles and Seattle.

Our findings are relevant to researchers seeking to develop low-cost and timely methods that allow for direct measurement of the built environment to study its association with obesity and other health outcomes. All the data and computational methods used in this study are openly available, allowing for comparisons of study results across regions with varying populations and geographies. In addition, our results are also relevant to people monitoring obesity prevalence or working to develop public health programs to decrease obesity. We show that models fitted solely to the features of the built environment can provide reasonable estimates of neighborhood obesity prevalence, which are typically delayed from official sources by several years. For this study, we used obesity prevalence estimates from 2014, because more recent values were unavailable. Ideally, methods and programs should focus on combining individual- and neighborhood-level data to provide timely estimates of neighborhood obesity prevalence.

### Limitations

This study has some limitations. First, the obesity prevalence estimates from the Behavioral Risk Factor Surveillance System are based on self-reported height and weight, which have been shown to be biased and tend to lead to lower estimates of obesity prevalence.^55,56^ In addition, BMI does not allow for the direct measurement of body fat, which can vary across sex, age, race, and ethnicity. Furthermore, mortality and morbidity risk may vary across different race and ethnicities at the same BMI.^57,58^ Differences also occur in the timing of the obesity data and the satellite images, which can introduce bias into our analysis.

Furthermore, we used a CNN that is trained for object recognition to extract features relevant to the built environment because of the lack of a labeled data set for high- and low-obesity areas. This approach puts some restrictions on the interpretability of features used in our model. A CNN trained to classify land use patterns (eg, the UC [University of California] Merced land use data set^59^) also encodes fine-grained information about the built environment. Thus, alternatively, we can use such a CNN trained on a large land-use data set as a fixed-feature extractor and examine the performance of these vectors for classifying high- and low-obesity areas. This CNN will allow us to associate land use patterns with obesity trends and lend greater interpretability to prediction models. However, we were unable to find openly available networks pretrained on aerial and satellite imagery data sets for such a task. Google Street View images are a potential resource for capturing community activity levels at greater precision. For example, presence of persons jogging in the neighborhood might be an indicator of lower obesity prevalence in the community.

## Conclusions

Our study provides evidence of the efficacy of CNNs at associating obesity prevalence with significant physical environment features and opens possibilities for refining the methods for a more consistent and useful application. To make our approach useful for public health and community planning efforts, our future efforts will focus on assessing disparities based on neighborhood racial composition and socioeconomic status. Socioeconomic status has been linked to obesity and other health outcomes. For example, Gordon-Larsen et al^21^ found that lower socioeconomic status blocks were more likely to have fewer physical activity facilities, and these disparities in access could be associated with differences in overweight patterns. Review studies focused on the African American population and disadvantaged populations highlighted a strong association between safety and physical activity and obesity, especially in urban regions.^38,60^ This finding could also explain the associations we noted between police stations and the high obesity prevalence in the POI data. These populations were also less likely to have access to needed resources and environments typically associated with lower obesity prevalence. Furthermore, longitudinal studies using our approach can assess changes in the built environment that may be associated with increases or decreases in neighborhood obesity prevalence.

Results in this study support the association between features of the built environment and obesity prevalence. Neighborhood-level interventions to encourage physical activity and increase access to healthy food outlets could be combined with individual-level interventions to aid in curbing the obesity epidemic.

## Notes

Supplement. **eMethods.** Materials and Data Analysis **eTable 1.** Places of Interest **eTable 2.** Demographic and Obesity Data **eFigure 1.** Illustration of Transfer Learning Approach **eFigure 2.** VGG-CNN-F Convolutional NN Architecture **eFigure 3.** t-SNE Visualization of Features Extracted From VGG-CNN-F for Satellite Imagery **eFigure 4.** Actual Obesity Prevalence and Cross-validated Model Estimates of Obesity Prevalence **eFigure 5.** Google Satellite Images for Seattle Showing Locations With Low and High Obesity Prevalence, Respectively **eFigure 6.** Actual Obesity Prevalence and Cross-validated Model Estimates of Obesity Prevalence **eFigure 7.** Google Satellite Images for San Antonio Showing Locations With Low and High Obesity Prevalence, Respectively **eFigure 8.** Actual Obesity Prevalence and Cross-validated Model Estimates of Obesity Prevalence **eFigure 9.** Google Satellite Images for Memphis Showing Locations With Low and High Obesity Prevalence, Respectively **eFigure 10.** Actual Obesity Prevalence and Cross-validated Model Estimates of Obesity Prevalence **eFigure 11.** Google Satellite Images for Los Angeles Showing Locations With Low and High Obesity Prevalence, Respectively **eFigure 12.** Out-of-Sample Predictions of Obesity Prevalence Plotted Against Actual Obesity Prevalence **eFigure 13.** Cross-validated Model Estimates of Per Capita Income Plotted Against Actual Per Capita Income **eFigure 14.** Out-of-Sample Model Predictions of Per Capita Income Plotted Against Actual Per Capita Income **eFigure 15.** Actual Per Capita Income and Cross-validated Model Estimates of Per Capita Income **eFigure 16.** Actual Per Capita Income and Cross-validated Model Estimates of Per Capita Income **eReferences.**
