# Supplementary material for: Use of Deep Learning to Examine the Association of the Built Environment With Prevalence of Neighborhood Adult Obesity
Source: JAMA Netw Open. 2018 Aug 31;1(4):e181535. doi: 10.1001/jamanetworkopen.2018.1535 (PMC6324519; doi:10.1001/jamanetworkopen.2018.1535)
Supplement: Supplement. — eMethods. Materials and Data Analysis eTable 1. Places of Interest eTable 2. Demographic and Obesity Data eFigure 1. Illustration of Transfer Learning Approach eFigure 2. VGG-CNN-F Convolutional NN Architecture eFigure 3. t-SNE Visualization of Features Extracted From VGG-CNN-F for Satellite Imagery eFigure 4. Actual Obesity Prevalence and Cross-validated Model Estimates of Obesity Prevalence eFigure 5. Google Satellite Images for Seattle Showing Locations With Low and High Obesity Prevalence, Respectively eFigure 6. Actual Obesity Prevalence and Cross-validated Model Estimates of Obesity Prevalence eFigure 7. Google Satellite Images for San Antonio Showing Locations With Low and High Obesity Prevalence, Respectively eFigure 8. Actual Obesity Prevalence and Cross-validated Model Estimates of Obesity Prevalence eFigure 9. Google Satellite Images for Memphis Showing Locations With Low and High Obesity Prevalence, Respectively eFigure 10. Actual Obesity Prevalence and Cross-validated Model Estimates of Obesity Prevalence eFigure 11. Google Satellite Images for Los Angeles Showing Locations With Low and High Obesity Prevalence, Respectively eFigure 12. Out-of-Sample Predictions of Obesity Prevalence Plotted Against Actual Obesity Prevalence eFigure 13. Cross-validated Model Estimates of Per Capita Income Plotted Against Actual Per Capita Income eFigure 14. Out-of-Sample Model Predictions of Per Capita Income Plotted Against Actual Per Capita Income eFigure 15. Actual Per Capita Income and Cross-validated Model Estimates of Per Capita Income eFigure 16. Actual Per Capita Income and Cross-validated Model Estimates of Per Capita Income eReferences. [file jamanetwopen-1-e181535-s001.pdf]

Supplementary Online Content

Maharana A, Nsoesie EO. Use of deep learning to examine the association of the built environment with prevalence of neighborhood adult obesity. *JAMA Netw Open.* 2018;1(4):e181535. doi:10.1001/jamanetworkopen.2018.1535

eMethods. Materials and Data Analysis
eTable 1. Places of Interest
eTable 2. Demographic and Obesity Data
eFigure 1. Illustration of Transfer Learning Approach
eFigure 2. VGG-CNN-F Convolutional NN Architecture
eFigure 3. t-SNE Visualization of Features Extracted From VGG-CNN-F for Satellite Imagery
eFigure 4. Actual Obesity Prevalence and Cross-validated Model Estimates of Obesity Prevalence
eFigure 5. Google Satellite Images for Seattle Showing Locations With Low and High Obesity Prevalence, Respectively
eFigure 6. Actual Obesity Prevalence and Cross-validated Model Estimates of Obesity Prevalence
eFigure 7. Google Satellite Images for San Antonio Showing Locations With Low and High Obesity Prevalence, Respectively
eFigure 8. Actual Obesity Prevalence and Cross-validated Model Estimates of Obesity Prevalence
eFigure 9. Google Satellite Images for Memphis Showing Locations With Low and High Obesity Prevalence, Respectively
eFigure 10. Actual Obesity Prevalence and Cross-validated Model Estimates of Obesity Prevalence
eFigure 11. Google Satellite Images for Los Angeles Showing Locations With Low and High Obesity Prevalence, Respectively
eFigure 12. Out-of-Sample Predictions of Obesity Prevalence Plotted Against Actual Obesity Prevalence
eFigure 13. Cross-validated Model Estimates of Per Capita Income Plotted Against Actual Per Capita Income
eFigure 14. Out-of-Sample Model Predictions of Per Capita Income Plotted Against Actual Per Capita Income
eFigure 15. Actual Per Capita Income and Cross-validated Model Estimates of Per Capita Income
eFigure 16. Actual Per Capita Income and Cross-validated Model Estimates of Per Capita Income
eReferences.

This supplementary material has been provided by the authors to give readers additional information about their work.

© Maharana A et al. *JAMA Network Open*.

© Maharana A et al. *JAMA Network Open*.

eMethods. Materials and Data Analysis

Data on Obesity Prevalence

We obtained 2014 estimates on annual crude obesity prevalence from the 500 cities project[1]. We used crude obesity prevalence estimates from 2014 because adjusted estimates and more recent annual estimates were unavailable. These obesity estimates are derived from data from the United States Centers for Disease Control and Prevention (CDC) Behavioral Risk Factor Surveillance System (BRFSS)[2]. BRFSS is an annual cross-sectional telephone survey used to measure behavioral risk factors of U.S. residents. The BRFSS includes all 50 U.S. states, the District of Columbia and U.S. territories (i.e., Puerto Rico, Guam and the Virgin Islands).

An individual is considered obese if their body mass index (BMI) is greater than or equal to 30 kg/m^2, where BMI is estimated from self-reported weight and height. Certain exclusions were applied, including removal of pregnant women and individuals reporting certain height and weight measurements[3]. The data includes respondents 18 years of age and older.

To illustrate our approach, we focused on estimates of obesity prevalence for census tracts in six cities – Los Angeles, California; Memphis, Tennessee; San Antonio, Texas; and Seattle, Tacoma and Bellevue, Washington. Because Seattle, Tacoma and Bellevue are neighboring cities with few census tracts, we combined their data into a single dataset, referred to as, Seattle. These cities were selected to reflect regions with varying obesity prevalence. Recent rankings of obesity prevalence by states lists Tennessee and Texas as sixth and eighth of fifty most obese

© Maharana A et al. *JAMA Network Open*.

states, respectively[4]. In contrast, the states of Washington and California have lower obesity rates and are ranked thirty-second and forty-seventh of fifty, respectively.

Satellite Imagery and Places of Interest Data

We downloaded recent satellite images for each census tract at the zoom level of 18 and image dimensions of 400 by 400 pixels (which was later resized to 224 x 224 for our analysis) from the freely available Google Static Maps API. Historical data matching the time period of the obesity estimates was unavailable. The satellite imagery data consisted of approximately 150,000 images, implying there were multiple images for each census tract. We also obtained a comprehensive list of places of interest (e.g., parks, restaurants, liquor stores, bus stations, night clubs) by performing a nearby search for each location in a square grid spanning a census tract, using the Google Nearby Search API. We included all categories of places of interest available through the API instead of focusing only on physical activity facilities, food and health locations which have been widely studied, because we reasoned that other categories could influence an individual's health behavior and physical activity frequency. For example, a high density of pet stores could indicate high pet ownership which could influence how often people go to parks and take walks around the neighborhood. Furthermore, the places of interest data varied by city, with some cities, such as Los Angeles, having finer classifications than others. The data was further cleaned to avoid duplicate counts of the same location. A comprehensive list of places of interest categories are included in the eTable 1.

© Maharana A et al. *JAMA Network Open*.

Deep Neural Network Model

Data extracted from satellite images have been used in several health-related applications ranging from infectious disease monitoring to estimating socioeconomic indicators such as, poverty[5-7]. To extract information from the 150,000 satellite images, we used a convolutional neural network (CNN), which is the state-of-art method for most computer vision tasks such as object recognition, scene labelling, image segmentation and pose estimation. More recently, CNNs have also been trained in image recognition for skin cancer, diagnosis of plant diseases and classification of urban landscapes[8-10].

To train a CNN from scratch to differentiate between regions with low and high obesity prevalence, we need a large corpus consisting of millions of labelled images. However, such training data is unavailable. Instead, we used a transfer learning approach (see eFigure 1), which involves applying a previously trained network to our dataset of unlabeled images to make inferences. We used a network, VGG-CNN-F, which has been previously trained (hereafter referred to as, pre-trained) for object recognition on the ILSVRC-2012 challenge dataset (subset of ImageNet database) and is freely available to the research community[11,14]. The trained network achieved 16.7% top-5 error on the challenge test set. This implies that the network is able to identify the correct class for every image within its top 5 predictions or predictions with highest probability. The network has helped achieve breakthroughs in other image recognition tasks through transfer learning.

The convolutional neural network consists of five convolutional layers and three fully connected layers. Each convolutional layer is composed of several two-dimensional filters which activate

the features required for classifying an object correctly. During training, the neural network learns to extract gradients, edges and patterns that aid in accurate object detection. The fully connected layers further process these features and convert them into single dimensional vectors. The output layer (final fully connected layer) is originally designed for classifying between 1000 object categories. Essentially, this neural network transforms a large two-dimensional image into a single vector of fixed dimension, containing only the most important descriptors of the image. This feature vector is extracted by deploying the network and making a forward pass through it for each image. It has been shown that these descriptors can be used with linear classifiers or regression models to perform tasks that are much different from object recognition[12]. We employ this technique to transform satellite images of dimension 224x224 into a feature vector of length 4096, taken from the seventh hidden layer or second fully-connected layer of the VGG-CNN-F network.

We also make a forward pass through the network for some images and examine the output maps from convolutional layers of the CNN, to see if built environment features are being highlighted by these filters and transmitted to the succeeding layers. The output maps are single channel images which can be plotted and compared to the original image for interpretation of the outputs. The results from this process are visualized in Figure 1.

To investigate whether our approach could differentiate between images from areas with low and high obesity prevalence, we extracted the 4096-dimensional feature vectors from the second fully connected layer of VGG-CNN-F for each image and projected these vectors onto a 2-dimensional space via t-distributed stochastic neighbor embedding (t-SNE). t-SNE is a non-

© Maharana A et al. *JAMA Network Open*.

linear dimensionality reduction algorithm which has been known to preserve local neighborhoods at the expense of global structure[15]. It can be implemented using various metrics for measuring distance between two vectors; we used the Euclidean distance. We also fit a 3-component Gaussian mixture model to tract-level obesity prevalence data in order to divide census tracts of a city into low, and high obesity areas. All images belonging to a high obesity census tract were tagged as high obesity areas for the purpose of visualization, similarly for low obesity census tracts. Further, the mean feature vector for a census tract is computed by taking the average of the vectors for all satellite images belonging to that particular census tract.

We observe well-formed clusters for low and high obesity prevalence in the visualization for San Antonio, showing that built environment features in these areas are distinct (eFigure 3). The Gaussian distribution means for census tracts in San Antonio with low and high obesity prevalence are 26.7 and 38.4, respectively. In contrast, the Gaussian distribution means for census tracts in Seattle-Tacoma-Bellevue with low and high obesity prevalence are 20.6 and 31.9 only, making Seattle an area with low obesity prevalence. This is reflected in the t-SNE visualization for Seattle - the high obesity labelled data points are outnumbered and do not form distinct clusters within themselves. The clustering of images in eFigure 3, demonstrates that our approach can differentiate between images from census tracts with low and high obesity prevalence.

Regression Modeling

We conducted three different sets of analyses. Our first aim was to quantify the association between the features of the built environment and obesity prevalence at the census tract level.

We assessed how much of the variation in obesity prevalence across all census tracts is explained by features of the built environment extracted from satellite images. Since the data contained a large number of features (n=4,096), we applied Elastic Net – a regularization and variable selection technique. A major benefit of Elastic Net is that it combines the advantages of Ridge regression and Least Absolute Shrinkage and Selection Operator (LASSO); insignificant covariates are eliminated, while correlated variables that are significant are maintained[13]. Next, we evaluated how well our model predicts obesity prevalence across all cities by splitting the data into two sets – a random sample representing sixty percent of the data for fitting and the remaining forty percent for model evaluation.

Our second aim was to quantify the association between the neighborhood density of places of interest (such as, fast food outlets and recreational facilities) to obesity prevalence. We used the same process for variable selection and regression modeling as previously described. We then compared the model coefficient of determination (R^2), root mean squared error (RMSE) and Pearson correlation between the actual and estimated observations to our previous results on estimating obesity using data on the built environment extracted from satellite images.

Lastly, we fit separate models to quantify how well the features of the built environment extracted from satellite images predict socioeconomic variables, such as per capita income. This analysis was undertaken because obesity prevalence tends to correlate with socioeconomic status and we wanted to investigate if this could partially explain the performance of our models in predicting obesity prevalence.

© Maharana A et al. *JAMA Network Open*.

We used the R statistical software for all the regression modeling[16]. Five-fold cross validation was applied to models for all regions except Memphis, for which we used a three-fold cross validation because the sample size was less than 200, which limits the number of data points used in each fold. To prevent over-fitting, we also limited the number of selected features to be less than or equal to the number of census of tracts.

eTable 1. Places of Interest

Accounting	Car Wash	Gas Station	Lodging	Police
Airport	Casino	General Contractor	Meal Delivery	Post Office
Amusement Park	Cemetery	Grocery or Supermarket	Meal Takeaway	Real Estate Agency
Aquarium	Church	Gym	Mosque	Restaurant
Art Gallery	City Hall	Hair Care	Movie Rental	Roofing Contractor
ATM	Clothing Store	Hardware Store	Movie Theater	RV Park
Bakery	Convenience Store	Health	Moving Company	School
Bank	Courthouse	Hindu Temple	Museum	Shoe Store
Bar	Dentist	Home Goods Store	Natural Feature	Shopping Mall
Beauty Salon	Department Store	Hospital	Neighborhood	Spa
Bicycle Store	Doctor	Insurance Agency	Night Club	Stadium
Book Store	Electrician	Jewelry Store	Painter	Storage
Bowling Alley	Electronics Store	Laundry	Park	Synagogue
Bus Station	Embassy	Lawyer	Parking	Taxi Stand
Cafe	Finance	Library	Pet Store	Train Station
Campground	Fire Station	Light Rail Station	Pharmacy	Transit Station
Car Dealer	Florist	Liquor Store	Physiotherapist	Travel Agency

© Maharana A et al. *JAMA Network Open*.

Car Rental	Funeral Home	Local Government Office	Place of Worship	University
Car Repair	Furniture Store	Locksmith	Plumber	Veterinary Care
				Zoo

© Maharana A et al. *JAMA Network Open*.

eTable 2. Demographic and Obesity Data

	Bellevue	Seattle	Tacoma	Los Angeles	Memphis	San Antonio
2010 Population	122,363	608,660	198,397	3,792,621	646,889	1,327,407
Median age (years)	38.5	36.1	35.1	34.1	33.0	32.7
18 years and over	96,410 (78.8%)	515,147 (84.6%)	152,760 (77.0%)	2,918,096 (76.9%)	478,921 (74.0%)	971,407 (73.2%)
Male	48,020 (39.2%)	256,561 (42.2%)	74,539 (37.6%)	1,441,341 (38.0%)	221,992 (34.3%)	466,126 (35.1%)
Female	48,390 (39.5%)	258,586 (42.5%)	78,221 (39.4%)	1,476,755 (38.9%)	256,929 (39.7%)	505,281 (38.1%)
Income (per capita, 2014)*	50,405	44,167	26,805	28,320	21,909	22,784
Obesity						
Crude prevalence	16.3 (95% CI, 16.2-16.3)	19.2 (95% CI, 19.2-19.3)	24.6 (95% CI, 24.6-24.7)	21.1 (95% CI, 21.1-21.1)	29.3 (95% CI, 29.2-29.4)	32.4 (95% CI, 32.4-32.5)
Age-adjusted prevalence	18.8 (95% CI, 18.6-18.9)	22.4 (95% CI, 22.3-22.5)	30.8 (95% CI, 30.6-31)	26.7 (95% CI, 26.7-26.8)	36.3 (95% CI, 36.2-36.5)	32.9 (95% CI, 32.8-32.9)

© Maharana A et al. *JAMA Network Open*.

*American Community Survey, 2014 5-year estimate.

eFigure 1. Illustration of Transfer Learning Approach

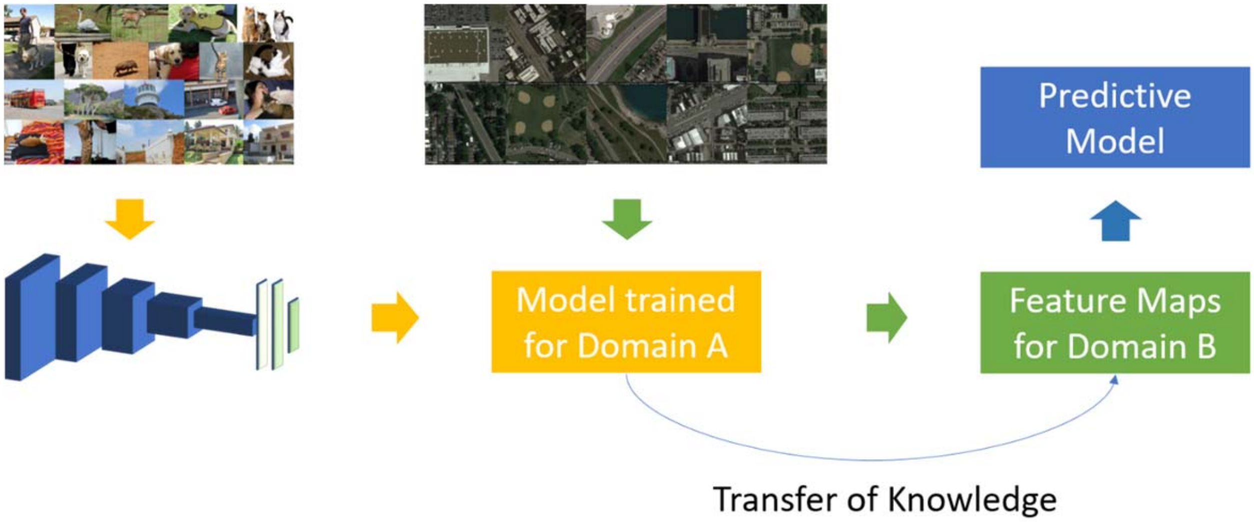

Let classification of objects from the ImageNet dataset be Domain A. The model trained for domain A has learnt how to interpret an image. We use this knowledge acquired by the model to understand satellite images. When the satellite images are fed as input to the model, we receive feature maps that encode this knowledge. These feature maps are used in a regression model to predict obesity values which is domain B. (Left composite image courtesy of ImageNet: http://image-net.org/)

© Maharana A et al. *JAMA Network Open*.

eFigure 2. VGG-CNN-F Convolutional NN Architecture

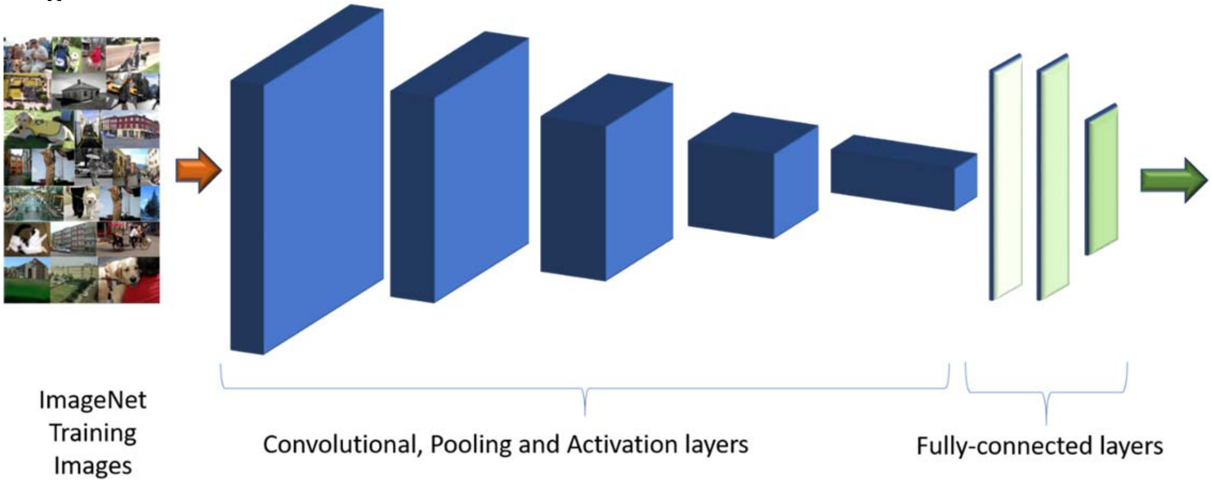

We have used the 8-layer VGG-CNN-F convolutional neural network for our project. In this architecture, the first five layers are a five-fold repetition of this arrangement: <convolutional layer->pooling layer->ReLU layer> Those layers are represented in blue. The next 3 layers (represented in green) are fully connected layers. (Left composite image courtesy of ImageNet: http://image-net.org/)

© Maharana A et al. *JAMA Network Open*.

eFigure 3. t-SNE Visualization of Features Extracted From VGG-CNN-F for Satellite Imagery

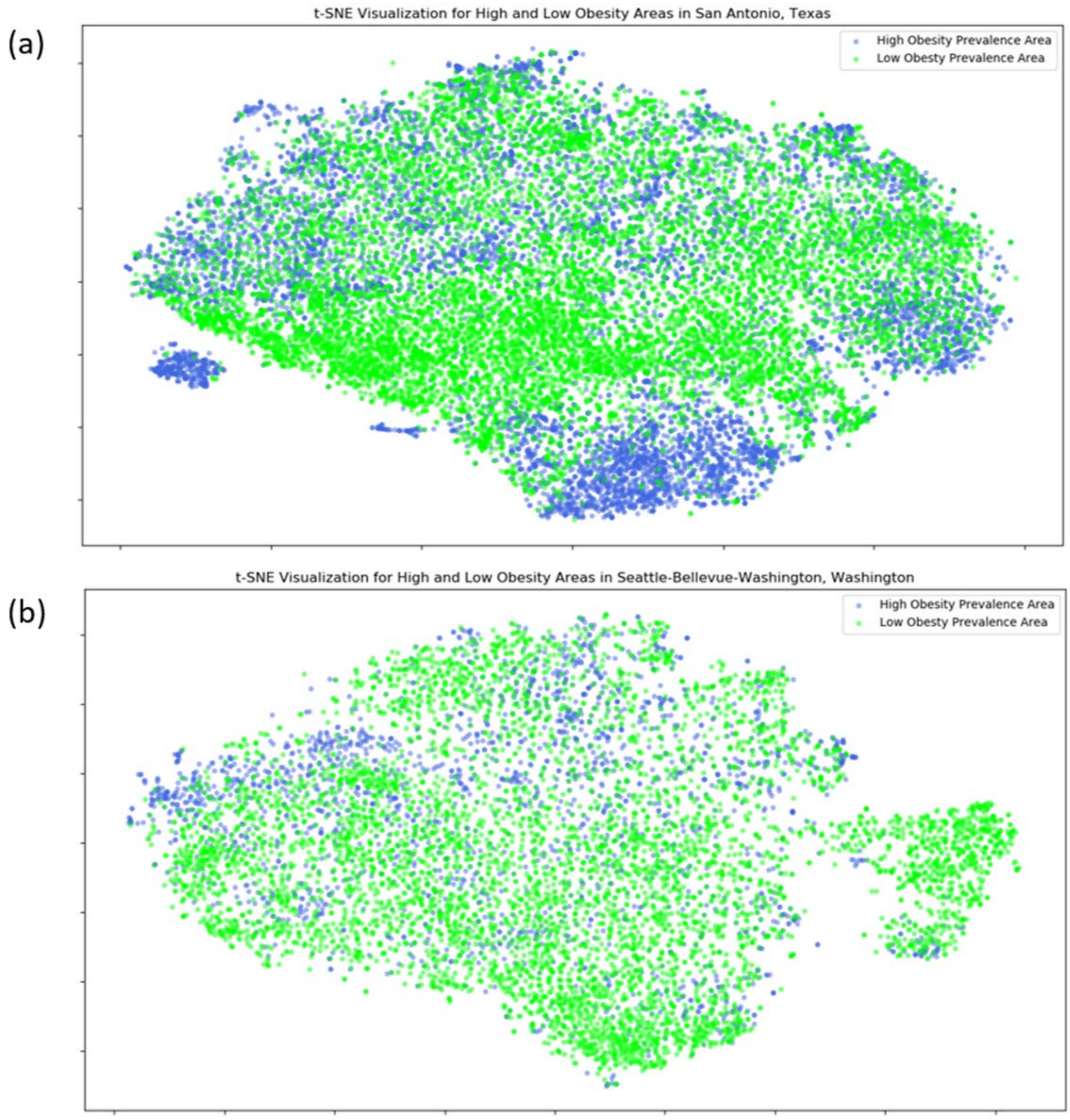

(a) San Antonio, Texas and (b) Seattle-Tacoma-Bellevue area, Washington.

© Maharana A et al. *JAMA Network Open*.

eFigure 4. Actual Obesity Prevalence and Cross-validated Model Estimates of Obesity Prevalence

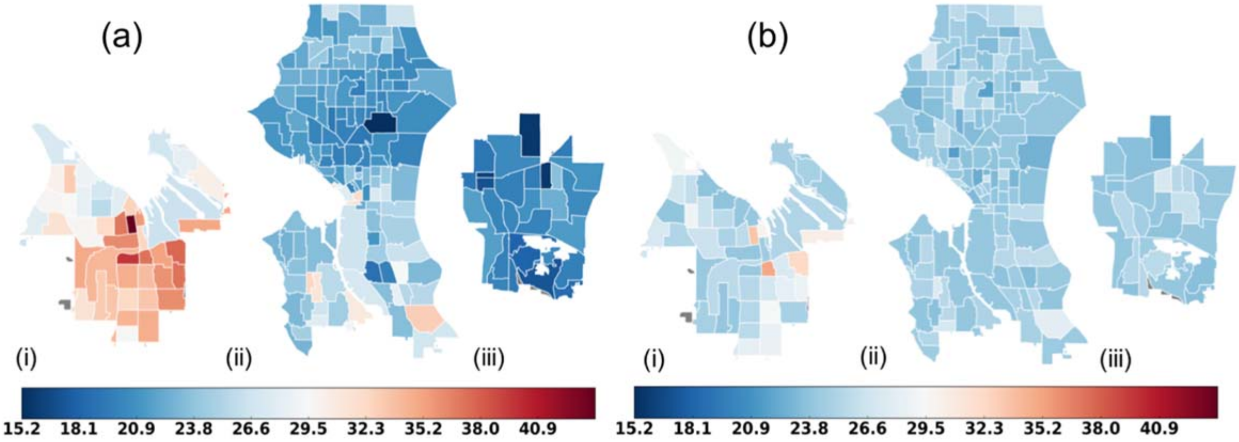

(a) actual and (b) cross-validated estimates of obesity prevalence for Bellevue (i), Seattle (ii) and Tacoma (iii), Washington based on the density of places of interest data. These cities are collectively referred to as Seattle in the manuscript. We do not have data for the gray shaded regions.

© Maharana A et al. *JAMA Network Open*.

eFigure 5. Google Satellite Images for Seattle Showing Locations With Low and High Obesity Prevalence, Respectively

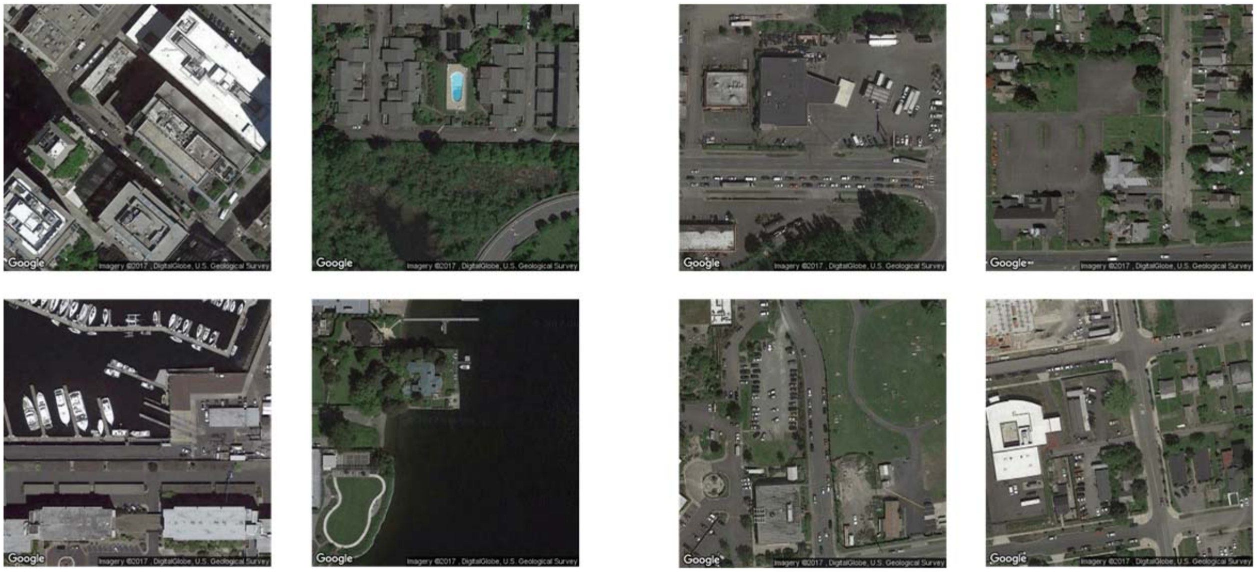

(Left grouping) Census tracts containing affluent neighborhoods (close to waterfront, swimming pool in apartment complex), waterfront businesses and downtowns are classified as low obesity areas in Seattle-Tacoma-Bellevue area. (Right grouping) Census tracts with small population density and less urbanized footprint are classified as high obesity areas in Seattle-Tacoma-Bellevue area.

© Maharana A et al. *JAMA Network Open*.

eFigure 6. Actual Obesity Prevalence and Cross-validated Model Estimates of Obesity Prevalence

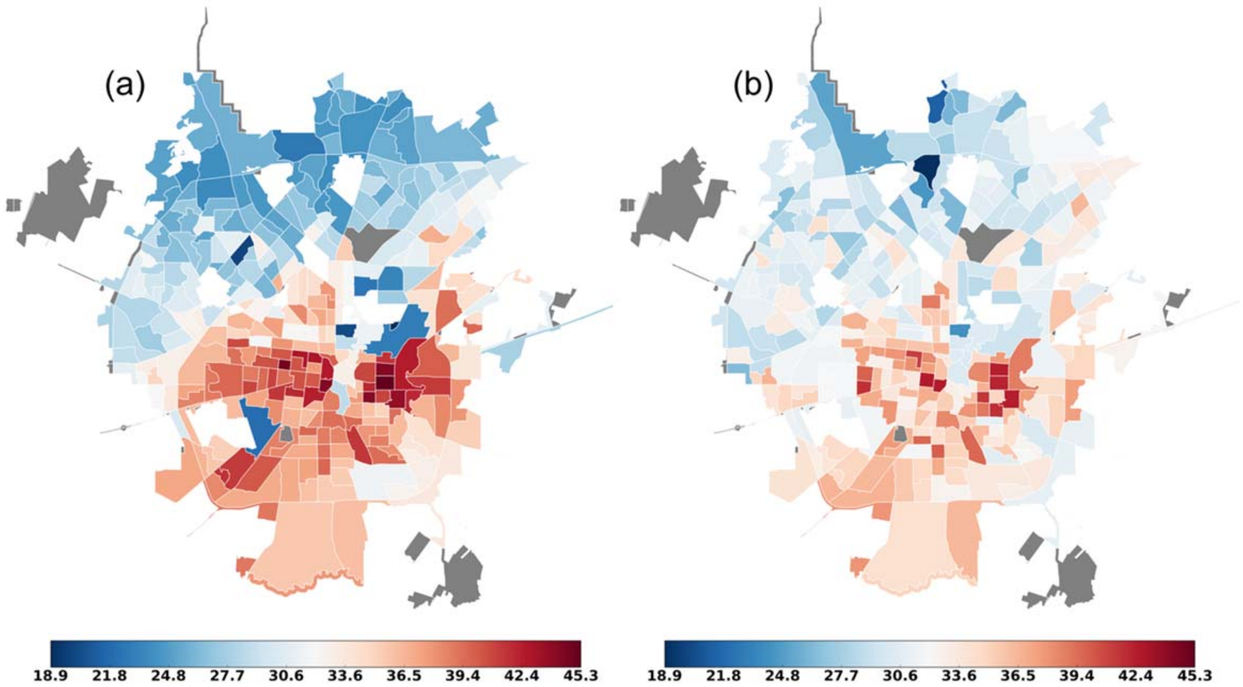

(a) actual and (b) cross-validated estimates of obesity prevalence for San Antonio, Texas based on the density of places of interest data. Unlike Seattle, the places of interest data appear to capture the variability in obesity prevalence across census tracts. We do not have data for the gray shaded regions.

© Maharana A et al. *JAMA Network Open*.

eFigure 7. Google Satellite Images for San Antonio Showing Locations With Low and High Obesity Prevalence, Respectively

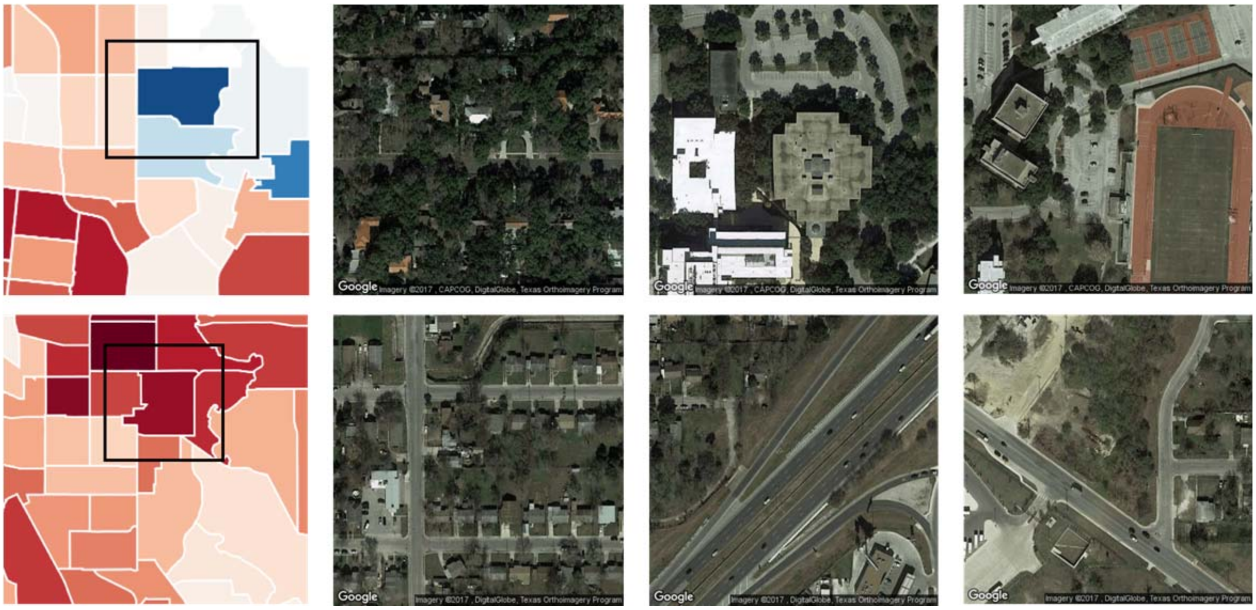

(Top Row) Census Tract 48029190400 – Images show dense green cover in residential areas and school building. (Bottom Row) Census Tract 48029130402 –The neighborhoods are dominated by larger roadways.

© Maharana A et al. *JAMA Network Open*.

eFigure 8. Actual Obesity Prevalence and Cross-validated Model Estimates of Obesity Prevalence

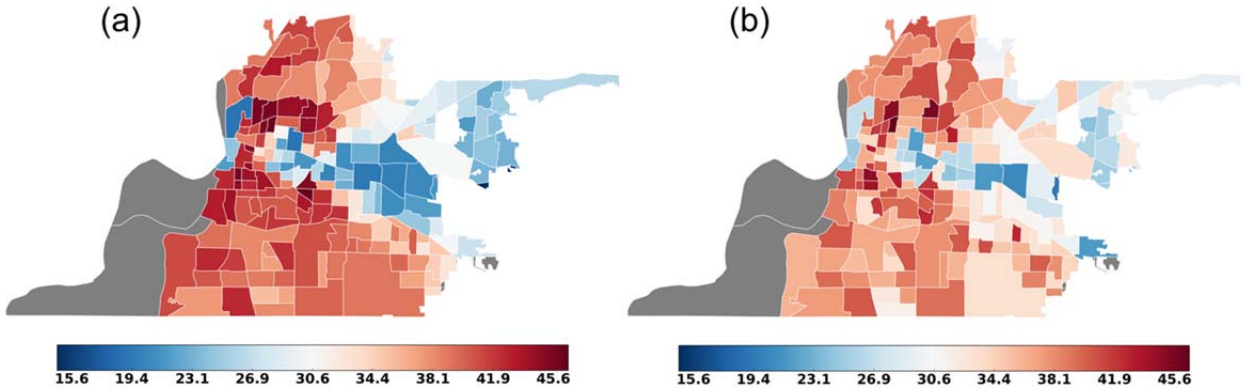

(a) actual and (b) cross-validated estimates of obesity prevalence for Memphis, Tennessee based on the density of places of interest data. The places of interest data capture the variability in neighborhood obesity prevalence for Memphis much better than it does for Seattle and Los Angeles (below), where the overall obesity prevalence is lower. We do not have data for the gray shaded regions.

© Maharana A et al. *JAMA Network Open*.

eFigure 9. Google Satellite Images for Memphis Showing Locations With Low and High Obesity Prevalence, Respectively

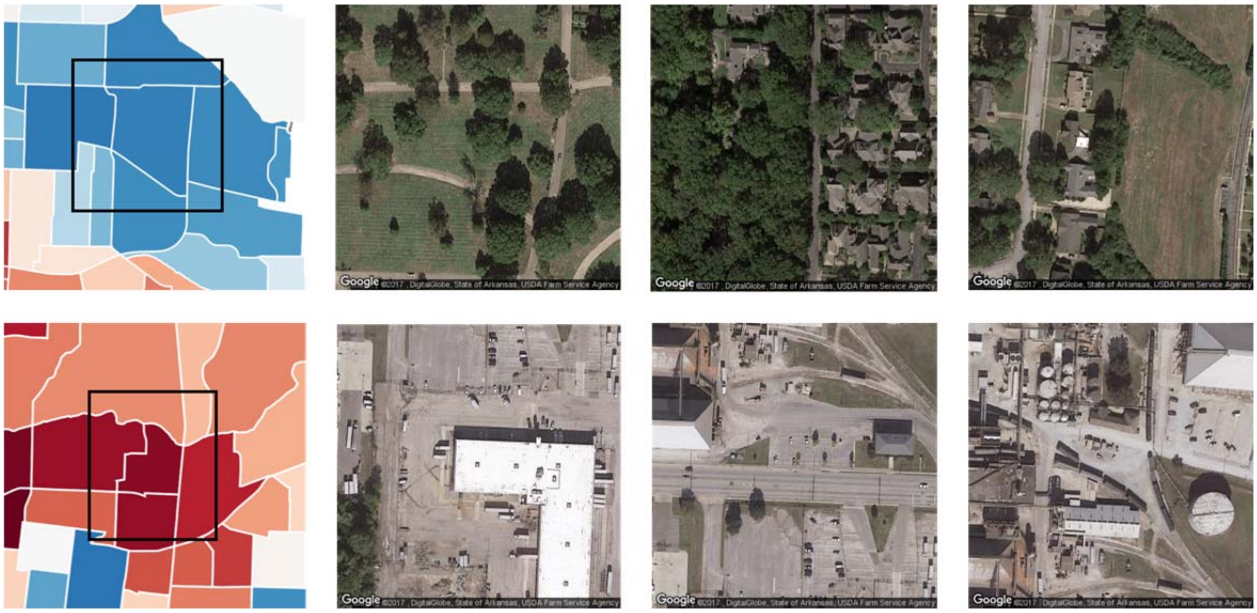

(Top Row) Census Tract 47157009600 – Images show green cover in neighborhood and walkways in field. (Bottom Row) Census Tract 47157000800 – Industrial area, presence of vehicles and sparse greenery.

eFigure 10. Actual Obesity Prevalence and Cross-validated Model Estimates of Obesity Prevalence

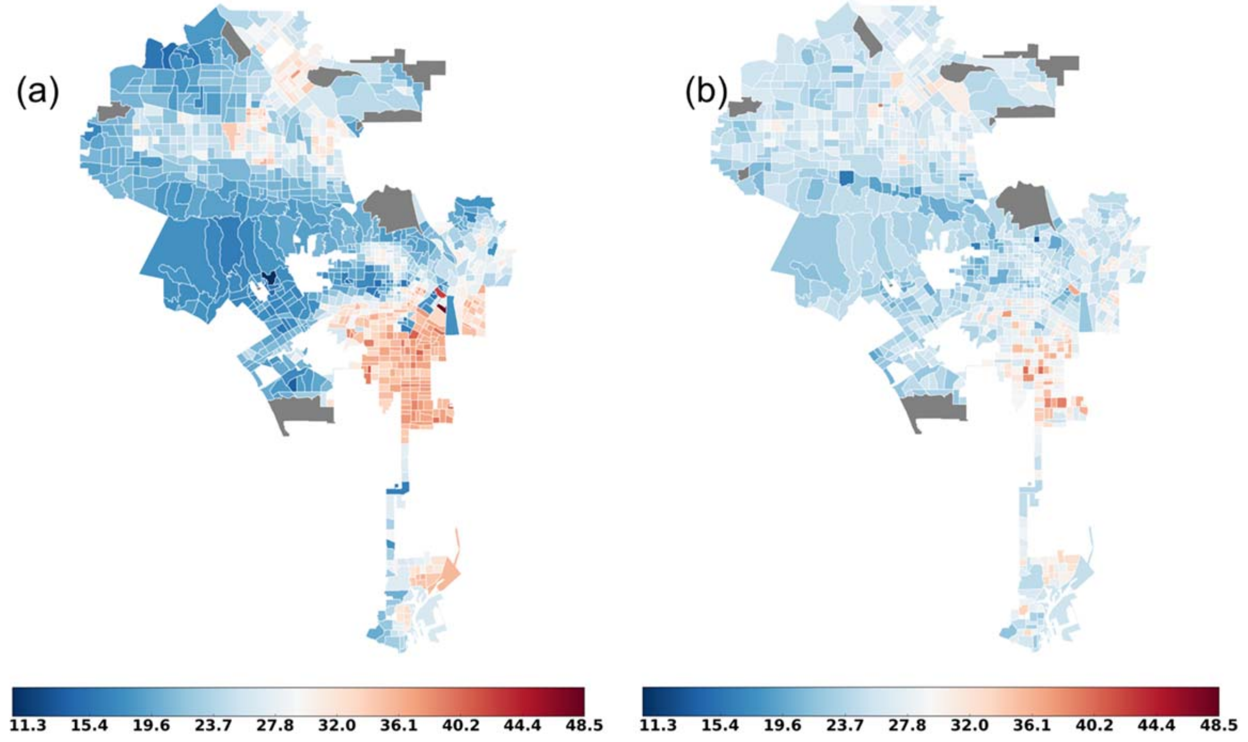

(a) actual and (b) cross-validated estimates of obesity prevalence for Los Angeles, California based on the density of places of interest data.

© Maharana A et al. *JAMA Network Open*.

eFigure 11. Google Satellite Images for Los Angeles Showing Locations With Low and High Obesity Prevalence, Respectively

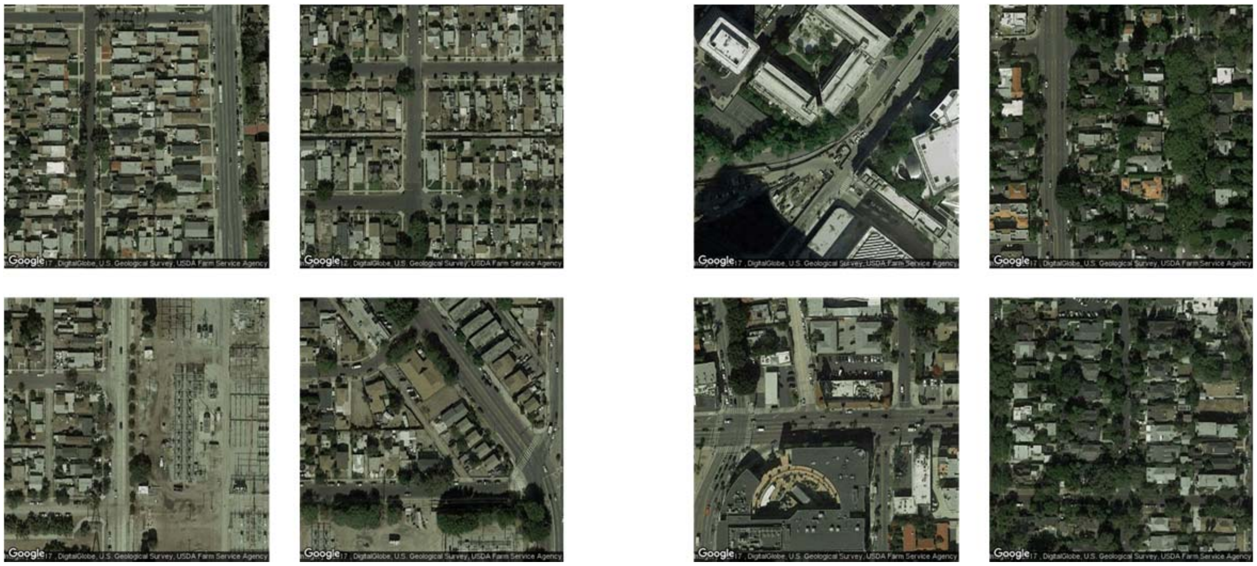

(Left grouping) High obesity census tracts are characterized by densely packed neighborhoods and less greenness. (Right grouping) Low obesity census tracts consist of mostly residential areas with more street greenness.

© Maharana A et al. *JAMA Network Open*.

eFigure 12. Out-of-Sample Predictions of Obesity Prevalence Plotted Against Actual Obesity Prevalence

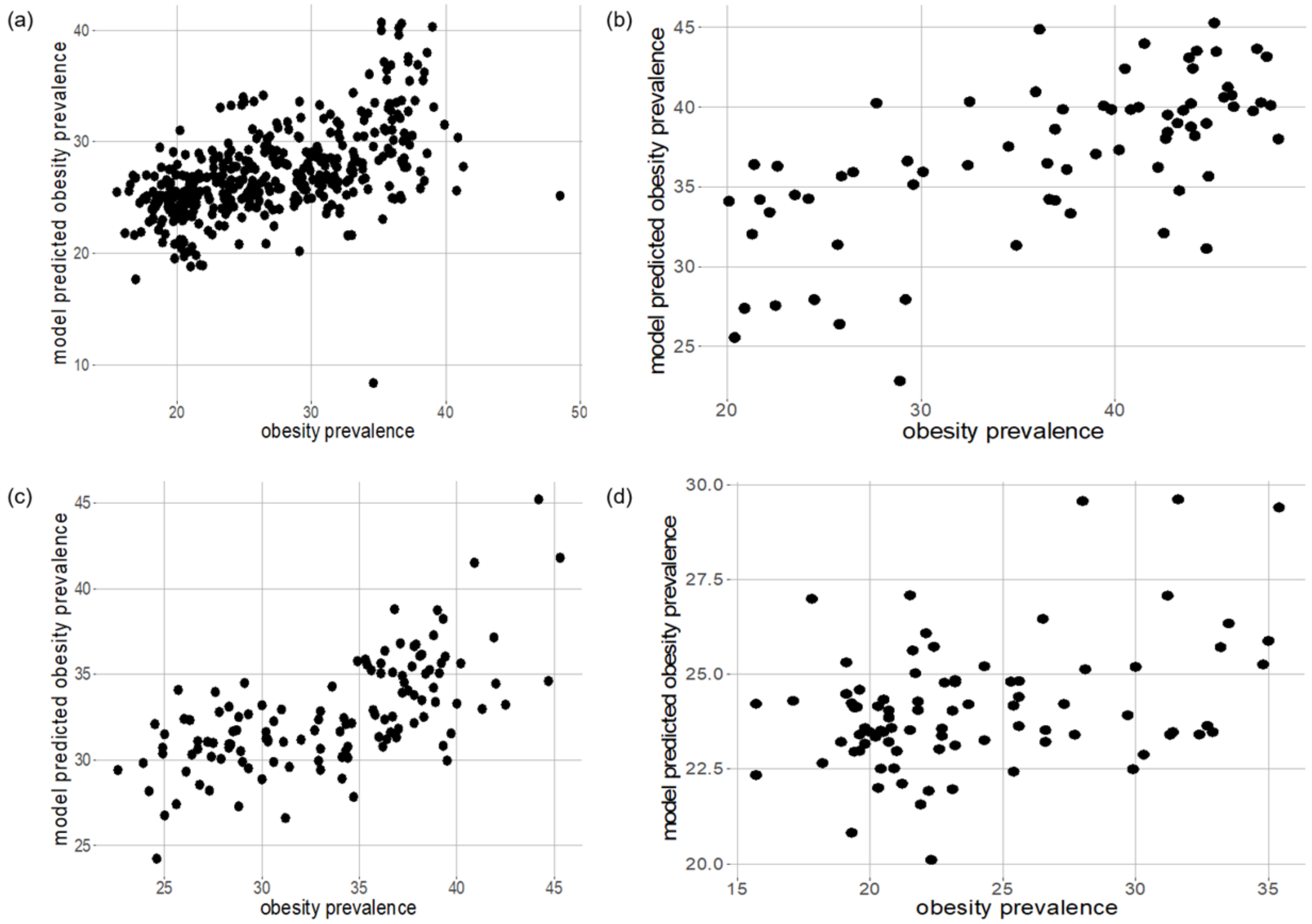

Obesity prevalence plotted against (a) Los Angeles, California (b) Memphis, Tennessee (c) San Antonio, Texas, (d) Seattle, Washington based on places of interest data.

© Maharana A et al. *JAMA Network Open*.

eFigure 13. Cross-validated Model Estimates of Per Capita Income Plotted Against Actual Per Capita Income

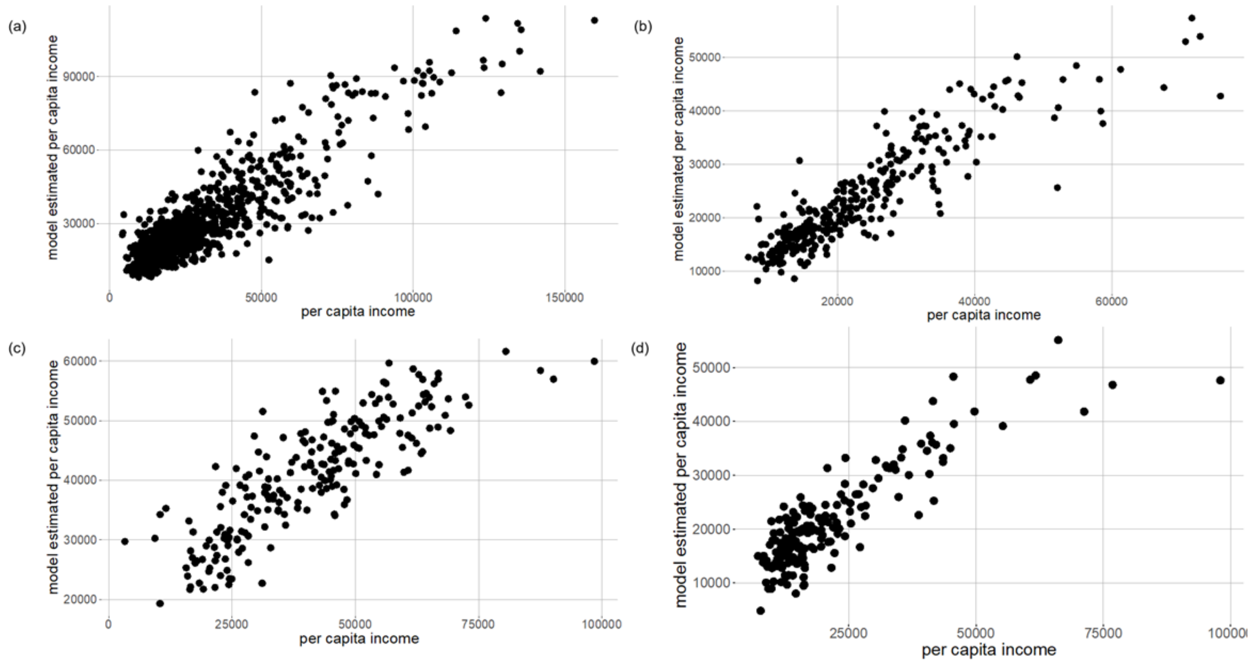

Actual per capita income for (a) Los Angeles, California (b) San Antonio, Texas (c) Seattle, Washington (d) Memphis, Tennessee

© Maharana A et al. *JAMA Network Open*.

eFigure 14. Out-of-Sample Model Predictions of Per Capita Income Plotted Against Actual Per Capita Income

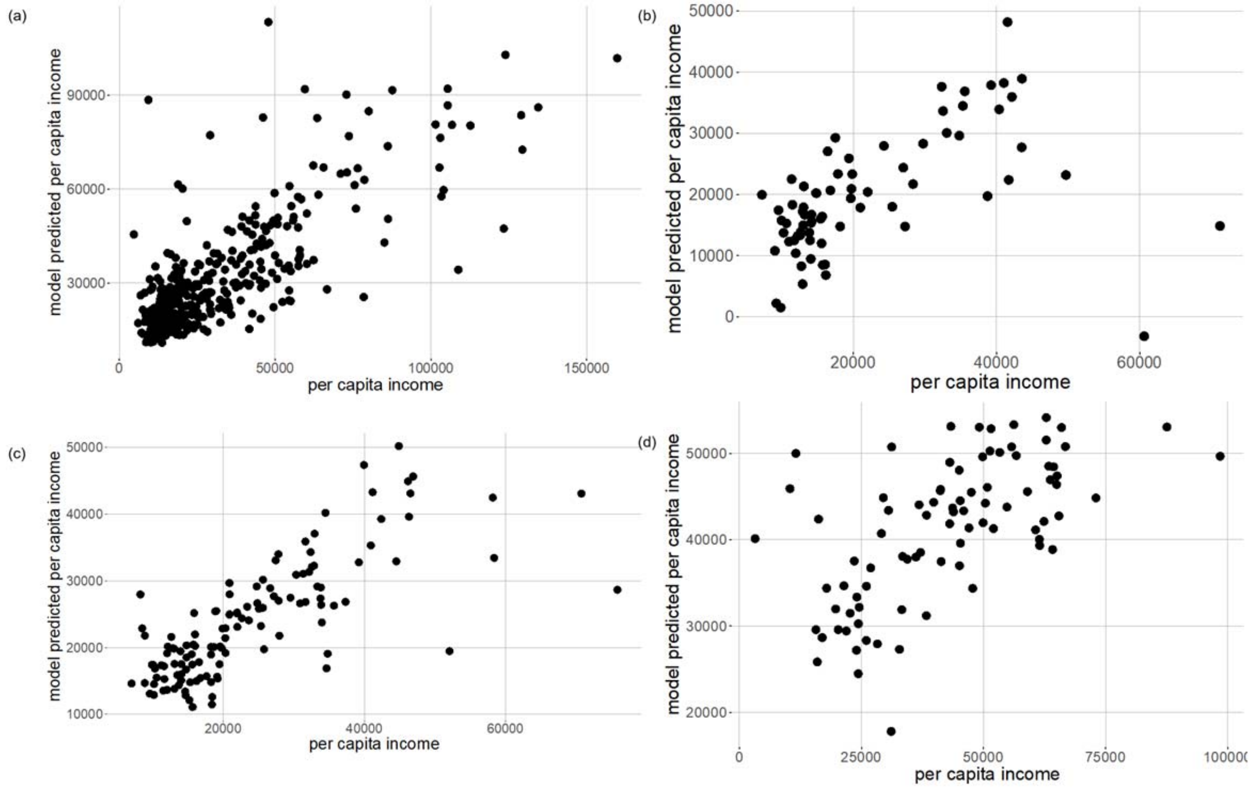

Plotted against actual per capita income for (a) Los Angeles, California (b) Memphis, Tennessee (c) San Antonio, Texas and (d) Seattle, Washington

© Maharana A et al. *JAMA Network Open*.

eFigure 15. Actual Per Capita Income and Cross-validated Model Estimates of Per Capita Income

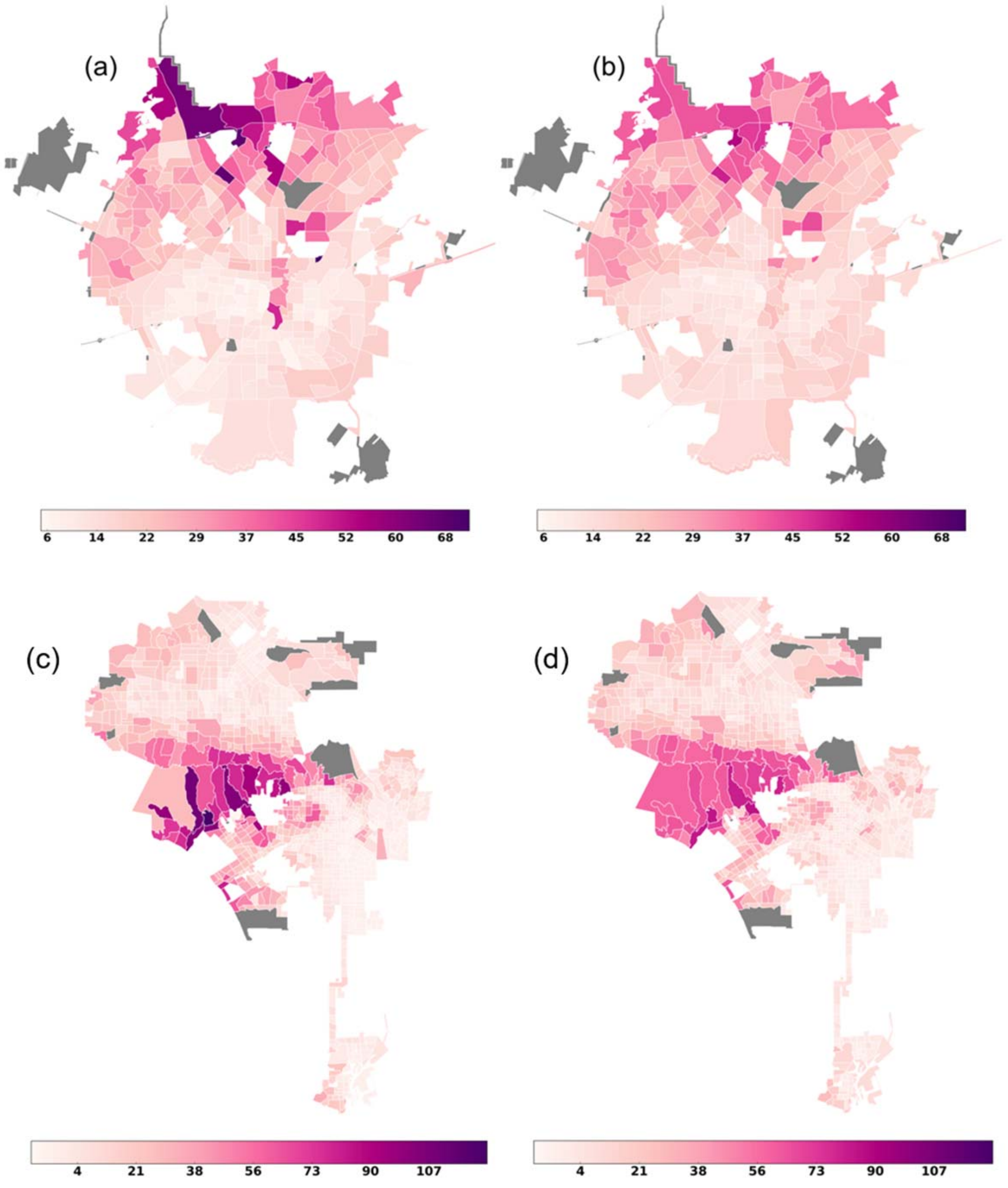

© Maharana A et al. *JAMA Network Open*.

(a), (c) actual and (b), (d) cross-validated estimates of per capita income for San Antonio, Texas and Los Angeles, California respectively, based on the features extracted from satellite images. The unit is in thousands of dollars.

© Maharana A et al. *JAMA Network Open*.

eFigure 16. Actual Per Capita Income and Cross-validated Model Estimates of Per Capita Income

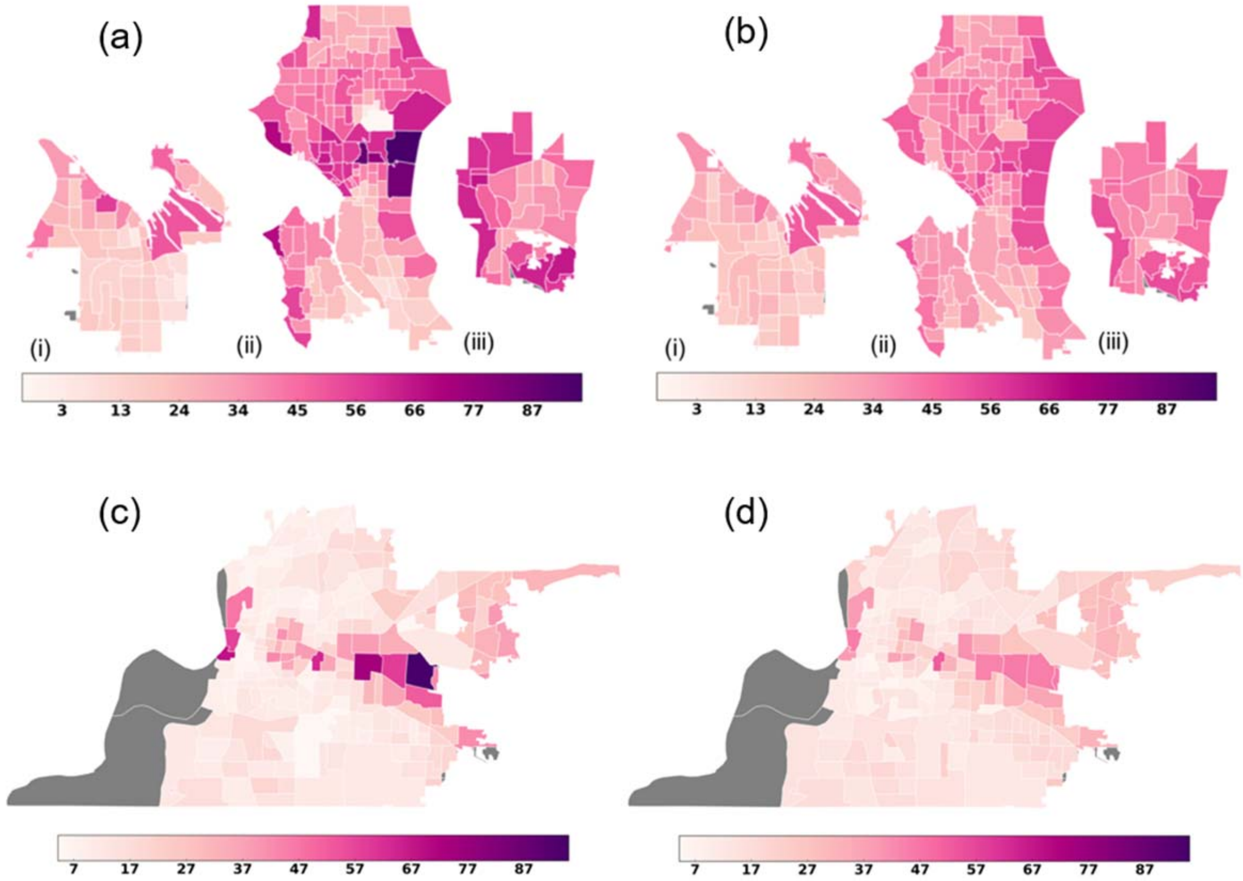

(a), (c) actual and (b), (d) cross-validated estimates of per capita income for Bellevue (i), Seattle (ii) and Tacoma (iii), Washington, and Memphis, Tennessee respectively, based on the features extracted from satellite images. The unit is in thousands of dollars.

© Maharana A et al. *JAMA Network Open*.

eReferences

1. CDC (2017) 500 Cities: Local Data for Better Health. Available at: https://www.cdc.gov/500cities/ [Accessed September 13, 2017].
2. CDC (2016) Overweight & Obesity: Adult Obesity Facts. Available at: https://www.cdc.gov/obesity/data/adult.html [Accessed May 19, 2017].
3. CDC (2016) 500 Cities: Local Data for Better Health. Unhealthy Behaviors. Available at: https://www.cdc.gov/500cities/definitions/unhealthy-behaviors.htm [Accessed September 13, 2017].
4. The State of Obesity. Obesity Rates & Trends - Adult Obesity in the United States (2017) Available at: https://stateofobesity.org/rates/ [Accessed October 14, 2017].
5. Nsoesie EO, Butler P, Ramakrishnan N, Mekaru SR, Brownstein JS (2015) Monitoring Disease Trends using Hospital Traffic Data from High Resolution Satellite Imagery: A Feasibility Study. *Sci Rep* 5:9112.
6. Karnieli A, Gilad U, Ponzet M, Svoray T, Mirzadinov R, Fedorina O (2008) Assessing land-cover change and degradation in the Central Asian deserts using satellite image processing and geostatistical methods. *J Arid Environ* 72(11):2093–2105.
7. Jean N, Burke M, Xie M, Davis WM, Lobell DB, Ermon S (2016) Combining satellite imagery and machine learning to predict poverty. *Science* 353(6301):790–794.
8. Mohanty SP, Hughes DP, Salathé M (2016) Using deep learning for image-based plant disease detection. *Front Plant Sci* 7.
9. Esteva A, Kuprel B, Novoa RA, Ko J, Swetter SM, Blau HM, Thrun S (2017) Dermatologist-level classification of skin cancer with deep neural networks. *Nature*

542(7639):115–118.

10. Albert A, Kaur J, Gonzalez M (2017) Using convolutional networks and satellite imagery to identify patterns in urban environments at a large scale. *ArXiv Prepr ArXiv170402965*.
11. Deng J, Dong W, Socher R, Li L-J, Li K, Fei-Fei L (2009) Imagenet: A large-scale hierarchical image database. *Computer Vision and Pattern Recognition, 2009. CVPR 2009. IEEE Conference on* (IEEE), pp 248–255.
12. Sharif Razavian A, Azizpour H, Sullivan J, Carlsson S (2014) CNN features off-the-shelf: an astounding baseline for recognition. *Proceedings of the IEEE Conference on Computer Vision and Pattern Recognition Workshops*, pp 806–813.
13. Friedman J, Hastie T, Tibshirani R (2001) *The elements of statistical learning* (Springer series in statistics Springer, Berlin).
14. Chatfield K, Simonyan K, Vedaldi A, Zisserman A (2014) Return of the devil in the details: Delving deep into convolutional nets. *ArXiv Prepr ArXiv 14053531*.
15. Maaten L van der, Hinton G. Visualizing data using t-SNE. *J Mach Learn Res*. 2008;9(Nov):2579–2605.
16. R Core Team (2013). R: A language and environment for statistical computing. R Foundation for Statistical Computing, Vienna, Austria. URL http://www.R-project.org/.

© Maharana A et al. *JAMA Network Open*.
